# CSO (Canadian Society of Otolaryngology - Head & Neck Surgery) position paper on rhinologic and skull base surgery during the COVID-19 pandemic

**DOI:** 10.1186/s40463-020-00476-9

**Published:** 2020-12-03

**Authors:** Yvonne Chan, Sarfaraz M. Banglawala, Christopher J. Chin, David W. J. Côté, Dustin Dalgorf, John R. de Almeida, Martin Desrosiers, Richard M. Gall, Artur Gevorgyan, A. Hassan Hassan, Arif Janjua, John M. Lee, Randy M. Leung, Bradford D. Mechor, Dominik Mertz, Eric Monteiro, Smriti Nayan, Brian Rotenberg, John Scott, Kristine A. Smith, Doron D. Sommer, Leigh Sowerby, Marc A. Tewfik, Andrew Thamboo, Allan Vescan, Ian J. Witterick

**Affiliations:** 1 Department of Otolaryngology - Head & Neck Surgery, University of Toronto, Toronto, ON Canada; 2 Division of Otolaryngology - Head and Neck Surgery, Department of Surgery, Dalhousie University, Saint John, NB Canada; 3 University of Montreal Hospital Center (CHUM) and Research Center (CRCHUM), Montreal, QC Canada; 4 Universite de Montreal, Montreal, QC Canada; 5 Department of Otolaryngology - Head and Neck Surgery, University of Manitoba, Winnipeg, MB Canada; 6 Department of Clinical Sciences, Northern Ontario School of Medicine, Thunder Bay, ON Canada; 7 Division of Otolaryngology - Head & Neck Surgery, University of British Columbia, Vancouver, BC Canada; 8 Calgary Sinus Centre, Calgary, AB Canada; 9 Division of Infectious Diseases, Department of Medicine, McMaster University, Hamilton, ON Canada; 10 Division of Otolaryngology - Head & Neck Surgery, Department of Surgery, McMaster University, Hamilton, ON Canada; 11 Department of Otolaryngology - Head and Neck Surgery, Western University, London, ON Canada; 12 Department of Otolaryngology - Head & Neck Surgery, Dalhousie University, Halifax, NS Canada; 13 Department of Otolaryngology - Head & Neck Surgery, McGill University, Montreal, QC Canada

## Abstract

Healthcare services in many countries have been partially or completely disrupted by the Coronavirus (COVID-19) pandemic since its onset in the end of 2019. Amongst the most impacted are the elective medical and surgical services in order to conserve resources to care for COVID-19 patients. As the number of infected patients decrease across Canada, elective surgeries are being restarted in a staged manner. Since Otolaryngologists – Head & Neck Surgeons manage surgical diseases of the upper aerodigestive tract where the highest viral load reside, it is imperative that these surgeries resume in a safe manner. The aim of this document is to compile the current best evidence available and provide expert consensus on the safe restart of rhinologic and skull base surgeries while discussing the pre-operative, intra-operative, and post-operative care and tips. Risk assessment, patient selection, case triage, and pre-operative COVID-19 testing will be analyzed and discussed. These guidelines will also consider the optimal use of personal protective equipment for specific cases, general and specific operative room precautions, and practical tips of intra-operative maneuvers to optimize patient and provider safety. Given that the literature surrounding COVID-19 is rapidly evolving, these recommendations will serve to start our specialty back into elective rhinologic surgeries over the next months and they may change as we learn more about this disease.

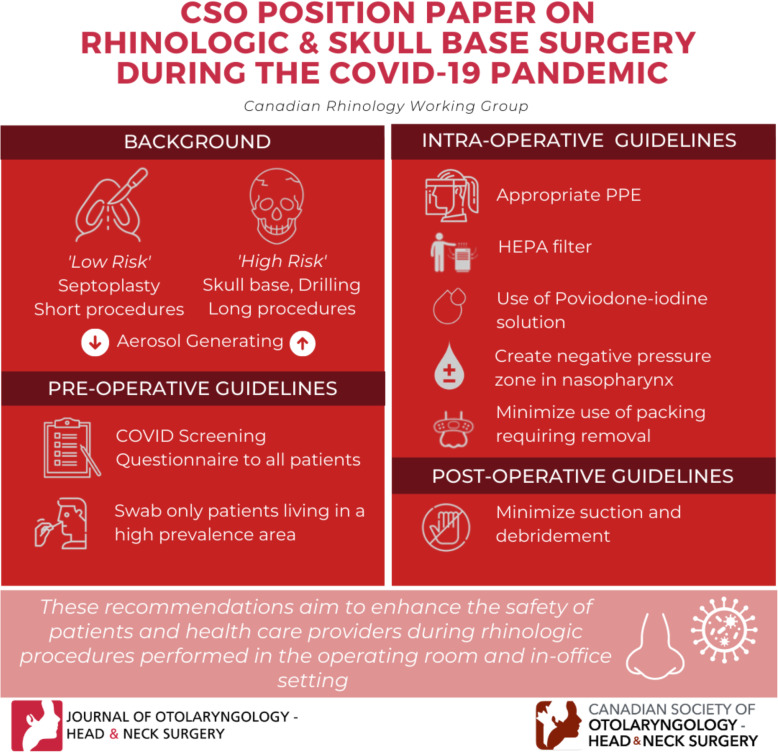

## Introduction and rationale

As the case numbers of COVID-19 have increased worldwide, it has become more apparent that the elevated viral load in the upper aerodigestive tract mucosa not only affects skull base surgery but essentially all diagnostic and therapeutic intranasal procedures that are routinely part of the scope of practice for Otolaryngologist - Head and Neck Surgeons [1]. A literature review of the current evidence regarding rhinologic surgery yielded several national position statements and recommendations. These were primarily composed of consensus expert opinion based on relatively small series as well as application from experiences with other similar diseases. The aim of this document is to summarize the current evidence and provide expert consensus recommendations surrounding the pre-operative, intra-operative, and post-operative care for rhinology and skull base patients in the setting of the SARS-CoV-2 (COVID-19) pandemic. As well, these recommendations endeavour to provide general principles ranging from triaging of cases, pre-operative COVID-19 screening, personal protection equipment (PPE) use to general and specific precautions in the operating room as well as practical tips of intra-operative maneuvers to optimize patient and provider safety while providing a high level of patient care.

The literature surrounding COVID-19 is evolving rapidly. These recommendations will serve as a “starter” document to get our specialty back into elective rhinologic surgery over the next weeks to months. As the epidemiology of the disease changes, these recommendations may change. This text represents a living document that will be updated over time. Please see the online version (https://www.entcanada.org/news-events/covid-19-alerts/) for the most up to date information. If substantial changes in clinical information arise, this taskforce will endeavour to keep this information updated periodically.

The guidelines and recommendations of each applicable regional health authority should be respected and checked regularly for updates. The information in this document is meant to be an adjunct to local recommendations, but not to supersede them.

## Timing of surgery and prioritization

There are currently no evidence-based recommendations for rhinologic surgical prioritization other than the French [2], American [3], and European [4] expert opinion guidelines. Depending on the rate of community spread, local infection and control measures, and availability of resources, it is recommended that previously scheduled cases be deferred until local circumstances allow for a return to more normal clinical activities. Consideration for urgent and emergent pathologies are outlined below and should be evaluated on a case-by-case basis. Patient factors such as COVID-19 positivity status, age, and associated comorbidities should also be taken into account when triaging these cases. For COVID-19 positive patients, we recommend those with emergent surgeries proceed with necessary PPE; urgent and semi-urgent surgeries may be delayed for a short time if feasible; and expedited and standard surgeries be delayed until the infection clears and the patient’s COVID-19 status is negative. The definition of a cleared COVID-19 infection may differ between health jurisdictions and regional health authority guidance should be followed (see Table 1).

**Table 1:** Recommended planning timeframes for various rhinologic surgeries during the COVID-19 era

Priority (Suggested Timeframe)	Surgical Procedure
Emergent (< 24 h)	- Lesion or disease process (e.g. mucocele, infection, inflammatory, neoplastic, bleed) with significant acute neurologic or ophthalmologic compromise
	- Invasive fungal sinusitis
	- Significant bleeding that cannot be managed by other means (e.g. packing, medical, interventional radiology)
Urgent (< 1 week)	- CSF leak repair (traumatic, iatrogenic)
	- Control of recurrent significant epistaxis (e.g. SPA ligation after failed nasal packing)
	- Severely displaced or open fracture
Semi-Urgent (< 4 weeks)	- Biopsy of concerning nasal mass
	- Malignant sinonasal/skull base skull tumour resection
Expedited (< 3 months)	- Benign nasal tumour (ex. Juvenile nasopharyngeal angiofibroma, hemangioma, inverting papilloma)
	- Mycetoma
	- Odontogenic sinusitis
	- CSF leak repair (spontaneous)
Standard (Regular Waitlist)	- Endoscopic sinus surgery for CRS or mucocele (without neurologic or ophthalmologic compromise)
	- Other rhinologic procedures (e.g. septoplasty, septorhinoplasty, dacrocystorhinostomy, orbital decompression, functional nasal surgery)
	- Skull base or orbital procedures for benign disease without neurologic compromise

## Pre-operative COVID-19 assessment

The most important step in the pre-operative assessment is a thorough screening of the patient, which includes questions about potential symptoms of COVID-19 as well as COVID-19 exposures. Symptomatic and known exposed patients who are asymptomatic should get tested for COVID-19 pre-procedure, and the procedure should optimally be postponed until 1) the test result is back, 2) a patient with significant exposure is no longer in self-isolation, and/or 3) symptoms have resolved. While the benefit of testing symptomatic patients is clear, the potential benefit of asymptomatic testing in the non-exposed and asymptomatic population is more controversial, and heavily depends on the local epidemiology and individual preferences of surgeons and patients.

Pre-operative COVID-19 testing has been highlighted as one of the important and necessary components of elective surgical reopening in a joint statement from the American College of Surgeons, American Society of Anesthesiologists, Association of Perioperative Registered Nurses and American Hospital Association [5, 6]. However, given issues with accessibility to tests, administrative challenges and limitations of locoregional laboratories, it is important to consider why pre-operative testing may be considered. The first consideration is whether a positive test would lead to a change in management. Changes in management can be: a) postponing the case if tested positive for either health care provider (HCP) or patient safety, or b) impact on the choice of PPE.

From a patient safety perspective, patients with a COVID-19 infection appear to have an increased risk of mortality following elective surgery [7]. In one study from Wuhan, the rate of ICU admission following elective surgery in asymptomatic COVID-19 positive patients was 44.1%, and the mortality rate was 20.5%, which was higher than the expected morbidity and mortality following these surgeries [7]. Significance of pre-operative COVID-19 testing is further highlighted by the high incidence of 30-day mortality of 38% in patients with peri-operative COVID-19 infection in an international study [8]. However, it remains unclear to what extent COVID-19 contributed to this higher than expected mortality rate given that the 7-day post-operative mortality was much lower, and to what extent the mortality could have been reduced by not operating on these patients. It also remains unclear to what extent these findings are generalizable to the surgical procedures discussed in this document as the vast majority of procedures were orthopedic and general surgical procedures, in regions with a high local prevalence of COVID-19.

Missing COVID-19 positives in screening that are infectious can result in harm to HCPs. COVID-19 is a highly infectious respiratory virus. During the SARS-CoV-1 outbreak in the early 2000s, aerosol generating medical procedures (AGMPs) were associated with an increased risk of viral transmission to HCPs [9]. In the case of SARS CoV-2, the nasal and oral cavities have an especially high viral load [1]. Many otolaryngologic surgeries involve manipulation of the upper aerodigestive tract, with an unknown risk of aerosol generation [9]. While the appropriate PPE for operating in COVID-19 positive patients has been debated, many sources have advocated for a powered air purifying respirator (PAPR) when surgery is necessary in COVID-19 positive patients [10]. However, this level of PPE is often unavailable within Canada, and requires training for safe donning and doffing. Similarly, in the post-operative phase, COVID-19 positive patients generate an increased risk of transmission to HCPs involved in their care, as well as other patients in the hospital.

This risk relates to asymptomatic individuals who screen negative for symptoms and exposures, but are pre-symptomatic within 48 h of onset of symptoms or currently asymptomatic carriers during the infectious period. These patients can be identified by pre-operative COVID-19 testing. Asymptomatic COVID-19 infections may contribute to a significant proportion of infections. Approximately 17% of the COVID-19 outbreak on the Diamond Princess cruise ship was made up of asymptomatic patients [11]. In Iceland, 50% of COVID-19 infections were asymptomatic [12]. Within Canada, a voluntary study in Calgary, AB screened asymptomatic participants and identified COVID-19 infections in 17.5% of the participants [13]. These studies emphasize that asymptomatic COVID-19 patients exist and are relatively common. In New York, asymptomatic testing during the peak of the pandemic for obstetrical patients discovered 14% of patients presenting for delivery were COVID-19 positive [14]. A study examining universal pre-operative testing in Alabama found 1% of patients scheduled for surgery tested positive for COVID-19 [5]. While the rates of asymptomatic carriers vary significantly, likely related to local disease prevalence, asymptomatic but infectious individuals can be an important source of transmission. But it is also important to emphasize that a positive test does not equate infectivity (see Section 3.2).

### Types of COVID-19 tests

As of June 7, 2020, there are twenty-two COVID-19 tests approved for use in Canada, with an additional thirty-three under review by Health Canada [15, 16]. Of the approved tests, 20 are lab-based tests, one is a point of care test approved for research only and one is both a point of care and lab-based test. Nineteen are nucleic acid technology based, utilizing reverse transcriptase polymerase chain reaction (RT-PCR) and 3 are serologic tests for immunoglobulins.

The RT-PCR tests are the predominant form of testing for the SARS-CoV-2 virus at this time. These are typically meant to be performed on specimens obtained from nasopharyngeal swabs, though oropharyngeal swabs, sputum, endotracheal specimens, and bronchoalveolar lavage samples can be used in some tests as well [5]. The current Center for Disease Control (CDC) approved test amplifies two separate genome regions. Where both regions are found, the test is positive. Where only one is found, the result is inconclusive, and where neither are present, then the test is negative [10].

The other set of tests that are emerging are the serological tests, which test for immunoglobulins, primarily IgM and IgG. These tests currently have no significant role in testing for active disease, and rather look for evidence of previous infection and immunity. Over time, serological testing will provide a better retrospective assessment of the true extent of the pandemic to date within Canada [17].

### Interpreting COVID-19 test results

RT-PCR has a specificity rate typically estimated to be in the range of 99–100% [18]. A high specificity means that a positive test can be generally considered a true positive in the context of a symptomatic patient in a community where COVID-19 is epidemic. The same would largely apply to an asymptomatic patient from a region with not-low prevalence of COVID-19, and especially during a local wave of infections. However, in an asymptomatic patient in a community with a low prevalence, the pre-test probability would be lower than the false-positive rate (1:100 to 1:1000 based on assumed specificity of 99–99.9%), as such there is a risk of finding multiple false-positives until one true-positive case can be identified. Furthermore, even a true-positive finding does not necessarily imply that a patient is infectious: it has been shown that infectivity declines significantly after onset of symptoms until day 8 in uncomplicated cases and that patients are less likely to be infectious thereafter; this is likely felt to be similar in asymptomatic individuals [19]. Therefore, a positive result can either be a pre-symptomatic or active/infectious asymptomatic carrier, a previously infectious but still PCR-positive individual, or a false-positive result. The latter two are responsible for the majority of positive results in a low prevalence setting, while the epidemiologic relevant/infectious cases are more likely the majority in a higher prevalence setting.

The sensitivity, quoted to be as low as 70% in the early stages of the pandemic based on studies from China, is reported to be as high 97% (92–100%) for nasopharyngeal swabs [18]. The viral testing sensitivity may also vary by assay and as such by laboratory to which the specimens are sent. Hence, key is to identify new and changing symptoms as part of the screening process as outlined above, and if there is still a doubt after one negative test, one can consider postponing the procedure, or to-retest the patient. When pre-operative testing identifies an asymptomatic COVID-19 positive patient in the setting of a reasonable high pre-test probability, surgery should be postponed if possible. The CDC has recommended these patients should be quarantined for a minimum of 7 days and be asymptomatic with two negative RT-PCR results performed 24 h apart to confirm clearance of SARS-CoV-2 before the surgery is rescheduled [10]. However, many Canadian jurisdictions have moved to a symptom-based clearance 14 days after onset of symptoms or positive test in asymptomatics. The main reason is the evidence that patients are no longer considered infectious after 14 days, and the fact that the PCR can remain positive for weeks or even months without implying infectivity.

The limitations are further compounded by the fact that the specificity and in particular sensitivity of the COVID-19 RT-PCR test in asymptomatic patients is less clear. This being said, the local prevalence of COVID-19 cases will substantially affect test interpretation based on pre-test probability, but also the surgical work. Regions with a low case prevalence will be in a different category, and should be more able to perform routine surgical work than regions with a high case prevalence.

### Who should receive pre-operative COVID-19 testing

Given the significant consequences of an unidentified COVID-19 positive patient undergoing operative care, universal pre-operative testing is recommended by many experts at this time [10]. This approach has been implemented in the majority of centres in North America during peak COVID-19 activity, and is recommended in the joint statement referenced above [20]. This is particularly important in patients undergoing otolaryngologic surgery, which may represent an additional risk to the OR staff. While the risk of aerosolization during upper aerodigestive surgery is unclear, and whether this potential aerosolization presents a significant risk to HCPs is also unclear, at this time one of the easiest ways to mitigate these concerns is to test patients pre-operatively in a non-low prevalence setting. With low and very low prevalence of COVID-19, the potential benefit can be outweighed by the potential harm of testing: false positives and non-infectious patients who are not infectious having to comply with quarantine, having their procedures unnecessarily delayed, patient thinking they have an active infection resulting in distress and unnecessary anxiety, as well as administrative inconveniences. With this in mind, surgeons and institutions must risk stratify and balance the pros and cons of pre-operative testing based on local epidemiology and the type and risk associated with surgery.

### Optimal pre-operative testing

Since the beginning of COVID-19 pandemic, there have been a number of recommendations produced by otolaryngology and rhinology communities worldwide. Uniformly, the recommendations include a thorough and comprehensive pre-operative COVID-19 symptom and risk factor questionnaire (e.g. one from Ontario [21]), and as well as at least a single COVID-19 test, typically a nasopharyngeal swabs for SARS-CoV-2 RT-PCR which has repeatedly been shown to have the highest sensitivity. Several groups have recommended an addition of chest imaging, such a chest x-ray [22] or CT chest [2, 23]. A study from Wuhan looking at correlation between chest CT and RT-PCR found chest CT to be more sensitive in detecting COVID-19 [24–26]. Others have recommended employing two RT-PCR testing in sequence, several days apart [27–29]. Finally, some have advocated for two RT-PCR tests, followed by chest imaging [30]. Given the more recent data, the sensitivity of NPS testing is much higher than originally reported, and given that the CT and chest x-ray findings are nonspecific, a single pre-operative NPS seems reasonable for asymptomatic patients who require testing. There is also a paucity of evidence to advocate for a more resource intensive protocol that included a CT scan or chest x-ray as recommended in some guidelines. The test is unable to identify a patient who is incubating the infection with minimal viral replication at the time of the test. Therefore, in an optimal setting, patients would receive the pre-operative nasopharyngeal swabs for the RT-PCR test 24(− 48) hours prior to surgery to limit the window of time during which the patient may be pre-symptomatic but not tested.

There have been many concerns about whether there are “high risk” otolaryngology surgeries which may warrant more comprehensive testing. Currently, there is no literature available to guide whether some surgeries require more testing than others. Recent publications suggest that surgeries involving drilling generate a significant amount of aerosols compared to surgeries without a drill. Surgeries involving lasers in the aerodigestive tract have a known risk of aerosol generation [31], and cautery may result in aerosolization into the surgical plume [32]. With the understanding that the respiratory mucosa harbours high levels of COVID-19 in infected individuals, this may increase the risk of nosocomial transmission in these patients. However, there is not a clear, definitive path to recommend additional testing in these patients.

### Special considerations

There are many additional factors that should be considered when assessing the need and practicality of pre-operative testing. Many health regions within Canada face logistical issues that affect the timeliness of the RT-PCR results. For example, most testing sites are not at the same location as the lab that performs the test. This necessitates pick up and delivery of the swabs from the testing site to the lab, which often means the lab will not receive the specimen until the day after it was performed. The RT-PCR test itself takes upwards of 6 h to be performed, not including the set up time or the interpretation time. The test also requires the appropriate reagents to be available, which were in short supply at the beginning of the pandemic. While turn-around times vary from region to region, the fastest test results are typically available in approximately 8 h. In areas where there is increased testing or increased travel times from testing sites to labs, additional time to receive results is required. It is important for the ordering physician to be familiar with the current wait times of the COVID-19 assay in their region, and request patients be tested as close to the procedure date as possible.

The testing of pediatric patients is another area for consideration. One must acknowledge that testing can be challenging in the pediatric population. Children are unsurprisingly the least compliant population with nasopharyngeal testing. It is still unclear whether children are more likely to remain entirely asymptomatic than adults, but clearly, paediatric patients are far less likely to develop severe infections. Hence, many children exhibit non-specific, albeit COVID-like symptoms at baseline, such as rhinorrhea. Careful screening for any even quite minor symptoms can help guide clinicians on the safety of proceeding with surgery in light of these considerations.

Further considerations in the pre- and post-operative period may include COVID-19 testing methodology. As rhinology patients may have intranasal pathology including crusting/infection, tumour, stents and skull base defects, they may be inappropriate subjects for nasal/nasopharyngeal swab testing. As such, alternate diagnostic strategies including sputum or oropharyngeal testing should be considered and discussed on a case-by-case basis in this patient population [33]. It is important, however, to consider the lower sensitivity of such testing.

A final consideration is whether to have patients isolate pre-operatively. In the early stages of surgical reopening, strict pre-operative quarantine of the patient and their household was implemented to varying degrees throughout Canada. As the case volume of COVID-19 has decreased, strict quarantine was reduced to isolation, then reduced to physical distancing and now in some regions, no pre-operative isolation is required. While the reason for pre-operative isolation is intuitive to many (to avoid exposure to the virus and provide a self monitoring period for symptoms), this comes at a great economic burden to most patients. It is preferable for patients to adhere to strict physical distancing, at the very least, as they await their surgery. Otherwise, pre-operative isolation should be determined by individual health regions based on their current caseload and degree of community spread, among other factors.

### COVID-19 pre-operative testing recommendation

1. Always test patients with new symptoms or changes in symptoms suggestive of a COVID-19 infection - regardless of local epidemiology.
2. In very low prevalence* areas, it is reasonable not to test for COVID-19 pre-operatively for rhinologic surgery.
3. In low prevalence* areas, consider pre-operative testing for COVID-19 status in high risk, aerosol generating rhinologic surgery (skull base surgery, drilling, long procedure > 1–2 h). For low risk rhinologic surgery (septoplasty, turbinate reduction, short procedures), it is reasonable not to test pre-operatively.
4. In high prevalence* areas, COVID-19 testing should be obtained if feasible pre-operatively for any rhinologic surgery.
5. If loco-regional prevalence not available, suggest default to pre-operative testing for any rhinologic surgery.

*Very low, low and high prevalence definitions vary. Please defer to your local health authority for active case levels to determine your loco-regional prevalence.

## General precautions in the operating room

As mentioned above, as a result of the substantial increase in surgical morbidity and mortality [8], as well as the significant resources that must be employed to ensure patient and healthcare worker safety in COVID-19 positive patients, identifying the COVID status of all patients is imperative. This is particularly important in those undergoing potentially high-risk AGMPs, such as rhinologic procedures. Still there will be some asymptomatic patients, who are asymptomatic and may falsely test negative, or were incubating when the specimen was obtained, and are unknowingly brought to the operating room. The frequency of this occurrence heavily depends on the local epidemiology as outlined above. Understanding that this may occur, systematic alterations that can be made in the operating suite, including configuration and room pressurization, as well as pre-operative medication strategies that may be employed in rhinology patients to minimize potential disease transmission.

### Operative suite configuration and negative pressure rooms

The single most important measure to reduce the risk of transmission to personnel in the ORs is minimizing the amount of personnel in the operating theater (particularly during AGMPs) [34, 35], and ensuring that the amount of thoroughfare is minimized during AGMPs, to reduce the potential risk of escape of bioaerosols into the surrounding areas [34].

Airflow principles in the operating room have become an important topic in minimizing risk of COVID-19 and any other respiratory pathogens to the operating room personnel. The majority of the operating rooms in Canada are considered ‘positive pressure’ relative to the surrounding hallways and corridors. The advantage of positive pressure is a purported reduction in surgical site infections (SSI), as the air is continuously pushed ‘out’ into the hallway, to prevent pathogens in the surrounding hallways from entering the operating theatre. Conversely, a ‘negative pressure’ room denotes a theatre for which air is continuously drawn into the room from the surrounding hallways [36]. The advantage of a negative pressure room is that, in a patient who may contaminate the room with infectious aerosols, there is less escape and dispersion of the aerosols to the rest of the surrounding hallways and rooms. This is felt to help prevent spread of pathogens beyond the operating theatre. The risk within the theatre, however, is only affected by the number of air cycles per hour regardless of whether the relative pressure is positive or negative. Other potential strategies to minimize aerosol dispersion risk are ensuring rooms have a high rate of air exchange, whether positive or negative pressure, and/or the use of portable High-Efficiency Particulate Air (HEPA) filters within positive pressure rooms [34].

The evidence regarding air pressurization in COVID-19 is limited. Many of these protocols are drawn from similar prior outbreaks, such as Severe Acute Respiratory Syndrome (SARS) and Middle East Respiratory Syndrome (MERS), as well as other pathogens like tuberculosis (TB). The majority of the guidelines support the use of negative pressure rooms. The ENT UK Epistaxis guidelines, The Italian Skull Base Society, and the International Head and Neck Scientific Group have all published on the recommendation for negative pressure operating theatres [34–36]. Liu et al. wrote an editorial [23] suggesting that negative pressure rooms are preferred as well for surgery in the COVID-19 positive patient; this is again based on an editorial in the anesthesia literature which states “…for aerosol generating medical procedures performed outside the operating theatre, a negative pressure/airborne isolation room with minimum 12 air changes (per hour) is preferred.” [37]

It should be noted that the recommendation for negative pressure is not universal. Couloigner et al. have recommended against negative pressure rooms in their article about COVID-19 and ENT surgery [38]. There are two main drawbacks to a negative pressure room. First, many hospitals do not have access to many, if any, negative pressure operating rooms, and as such it is not realistic to do all elective potentially aerosol generating surgeries in this setting. Secondly, theoretically the negative pressure room increases surgical site infections (SSI) by drawing pathogens into the operating theatre from the surrounding areas. However, in the head and neck, SSI from bacterial airborne contamination are very rare due in large part to the robust vascular supply.

Beyond the pressure settings in the operating room, there are other considerations that must be taken into account in order to optimize patient flow. In March 2020, Ti et al. described their operative suite setup of a negative pressure room for suspected or confirmed cases of COVID-19 [39]. It constitutes having a negative pressure room in the corner of the operating complex, connected to the rest of the OR facilities by a negative pressure ante-room. Many facilities do not have the option to choose which theaters are negative pressure capable, if any.

#### Operating room recommendations

In summary, based on our consensus and the majority of the recommendations in the literature, our group recommends using a negative pressure room for AGMPs in the rare instances when a positive or suspected COVID-19 case requires emergent or urgent rhinologic surgery. A HEPA filter can be used to reduce the risk if no negative-pressure OR is available for these patients. Negative-pressure ORs are not required or do not need prioritization for screen negative patients (with or without testing). Regardless of the direction of the pressure, the frequency of air exchanges in the OR should be maximized. Furthermore, the use of strategies to create a local negative pressure environment within the nose (see section 6.3 and 6.4) or in close proximity to the patient (for example via the use of portable HEPA filter units, or smoke evacuation tubing) may be beneficial.

It should be noted that these recommendations are based largely on consensus statement and expert opinion, and not on firm, experimental data.

### Pre-operative preparation for Rhinologic procedures

#### Pre-operative medications

Pre-operative systemic antibiotics and/or systemic steroids are frequently prescribed for rhinology patients to control underlying inflammation and to improve surgical visualization and outcomes.

International consensus guidelines suggest that pre-operative antibiotics are useful when a chronic rhinosinusitis patient’s clinical presentation indicates a potential for increased bleeding and longer surgery [40, 41]. There currently is no literature or opinion suggesting that this pre-operative antibiotics practice should change for COVID-19 patients.

The evidence supporting the benefits of pre-operative topical and systemic steroids prior to sinus surgery is well-established [40, 42, 43]. The risk of infectious (viral/bacterial/fungal) complications of glucocorticosteroid was not shown to be increased with a cumulative dose < 700 mg prednisone in a meta-analysis. In H1N1-related pneumonia patients, researchers demonstrated that the immunosuppression caused by systemic steroids was associated with an increased morbidity, mortality, and prolonged viral replication [44]. However, in a systematic review examining the safety and efficacy of systemic steroids for COVID-19 infections, as well as other coronavirus infections, influenza, community acquired pneumonias and acute respiratory distress syndromes, there is evidence that systemic steroids may reduce mortality in *severe* COVID-19 disease [45, 46]. A further high profile clinical trial has supported this idea, in that dexamethasone treatment was shown to decrease mortality in severe presentations of COVID-19 [47]. The effect of pre-operative systemic steroids for the asymptomatic COVID-19 patient needs further clarification, but at this point it appears that the potential benefit outweighs the potential risk.

Unlike systemic steroids, which may suppress the innate immunity resulting in the potential for increased viral replication, topical steroids are expected to have dual functions in blocking viral replications and host inflammation [48]. Multiple studies examining topical and inhaled steroids in viral infections confirm that they reduce the production of cytokines and monokines, including IL-8 and IL-6, in bronchial epithelial cells [49]. As a result, the authors do not believe that the practice of the routine pre-operative topical nasal steroids should change in the COVID-19 era.

#### Immediate pre-procedural nasal preparation (day of surgery)

Prior to the coronavirus pandemic, the use of endonasal antiseptics was not routine in rhinologic and skull base surgery since it has no preventative effect on post-operative infections [50]. During the COVID-19 era, their use has been revisited as a means of decreasing nasal viral load and preventing potential viral spread during trans-nasal surgery.

The enveloped SARS-CoV2 virus is known to survive in different environmental conditions (e.g. temperature, humidity) and on different surfaces found throughout the hospital - including aluminum, sterile sponges, latex surgical gloves, and biological fluids [51]. The knowledge that the nasal cavity and nasopharynx may act as a substantial reservoir for the virus has led surgeons to investigate the use of several virucidal strategies prior to nasal surgery. The efficacy of these nasal ‘disinfectant’ strategies must be evaluated in concert with their potential for toxicity to the nasal mucosa. Most of the tested surface or skin disinfectants in SARS-CoV2 are alcohol-based solutions (isopropanol or ethanol) [52], which are not safe for the nose.

Povidone-iodine (PVP-I) is a broad-spectrum antiseptic agent. It is widely available in multiple preparations, marketed for skin and mucosa, for the prevention of wound infections. PVP-I delivers free iodine molecules which have antimicrobial properties. In an in vitro study, Eggers et al. demonstrated that PVP-I is virucidal against the SARS-CoV-1 and Middle East Respiratory Syndrome Coronavirus (MERS-CoV) [53]. In their paper, these viruses were inactivated when exposed to concentrations as low as 0.23%, for as little as 15 s [53]. Ramenzapour et al’s in vitro study demonstrated that PVP-I at a concentration of 0.5% could be safely applied to cells of the nasal epithelium for 5 min with no toxicity, paracellular permeability, or ciliary beat frequency effects [54]. However, mucosal contact for 30 min was found to alter the transepithelial electrical resistance (TEER), still without changes to cilia beat frequency [54]. In another in vitro study, PVP-I was found to be ciliotoxic to respiratory epithelial cells at concentrations higher than 5%, reducing ciliary beat frequency to zero [55]. It appears we must be cautious when using topical PVP-I at either high concentrations (> 0.5%) or prolonged duration (> 5 min) as these have been shown to negatively affect the epithelium of the respiratory tract. To our knowledge, there are no in vivo or in vitro publications that demonstrate that PVP-I can safely eliminate SARS-CoV-2. Nevertheless, given the evidence of virucidal activity against other coronaviruses and the in vitro safety data above, some of the authors of this publication have employed a prophylactic protocol immediately pre-operatively (after intubation), in an attempt to decrease any potential nasal viral load. This protocol consists of diluting a commercial available Betadine USP 10% solution 1:20 to 0.5% and rinsing the nasal cavity for 15 s prior to any mucosal cuts being made in the OR. Of note, the authors also suggest placement of a throat pack to ensure no betadine exposure to the lower respiratory tract and subsequent nasal irrigation with saline to ensure dilution of any small volume betadine solution retained in the nasal cavities.

Antimicrobial photodisinfection therapy (aPDT) causes bacterial and viral cell destruction through oxidative disruption of the microbial cell membranes following absorption of photons in the presence of a photosensitizer. This technology has been employed to destroy microbes (*Staphylococcus aureus*) that may colonize the nose prior to procedures prone to surgical site infections, such as joint replacements [54]. Although aPDT is well-established, there are few reports about its possible application in respiratory infections [56]. The nasal viral load of COVID-19 makes it tempting to further explore the efficacy of aPDT as a preventive tool [57].

## Personal protection equipment (PPE) for operating room personnel

Personal Protective Equipment is an essential aspect of providing safe surgical care. There are several primary guiding principles for determining the use of PPE in surgery. These considerations include the safety and well-being of HCPs, patient safety, risk of the procedure to transmit COVID-19 from an infected patient to HCP, the possible risk of a patient having COVID-19 and community incidence of COVID-19. Other priorities include adherence to the regional, provincial and national guidelines regarding PPE use, the PPE stock and resupply available at an institution as well as the institutional access to COVID-19 testing. Internationally, a number of specialty societies have published recommendations for PPE as it relates specifically to endoscopic rhinologic and skull base surgeries during the COVID-19 pandemic including the European Rhinologic Society [58], the Italian Skull Base Society [27], the French Society of Otorhinolaryngology, the French College of Otorhinolaryngology, the French Syndicate of ENT Specialists [38], Young-Otolaryngologists of the International Federations of Oto-rhinolaryngological Societies [29], and the American Academy of Otolaryngology - Head and Neck Surgery (AAO-HNS) [3, 59].

### Protective equipment

#### Face mask

a loose-fitting device that creates a physical barrier between the mouth and nose of the wearer and potential contaminants in the immediate environment. It is fluid resistant and provides the wearer protection against large-particle droplets, splashes, sprays, or splatter that may contain microorganisms. It filtrates 3 μm particles [60]. Surgical masks are not typically reusable.

#### Respirator

is a respiratory protective device designed to achieve a very close facial fit and very efficient filtration of droplets and airborne particles. It reduces the wearer’s exposure to particles including small particle aerosols and large droplets. The type of respirator that we commonly use in medicine is particulate respirator. These can be further divided into (1) disposable or filtering facepiece respirators, where the entire respirator is discarded when it becomes unsuitable for further use; (2) reusable or elastomeric respirators, where the facepiece is cleaned and reused but the filter cartridges are discarded and replaced when they become unsuitable for further use; and (3) powered air purifying respirators (PAPRs), where a battery-powered blower moves the air flow through the filters. Respirators in this family is rated N, R, or P for its protection against oils. “N” respirators are not resistant to oil, “R” respirators are somewhat resistant to oil, and “P” respirators are oil proof. These respirators range from a filtration level of 95 to 100% [60].

##### N95 respirator

is the most commonly available disposable respirator. It filters out at least 95% of airborne particles including large and small particles 0.3 μm or larger. N95 respirators rely on fit testing and avoidance of mask manipulation during wear. Notably at least 10–15% of healthcare workers are unable to be suitably fitted with available N95 respirator options [61]. Poor fit testing may be related to facial physique, the length of a procedure, and need for specific positioning of the head during procedures for visualization [60].

##### Elastomeric respirator

is a half- or full-face mask made of soft rubber which can be disinfected and reused. Its filtration capacity is determined by the filter attached and ranges from N95 to P100 levels. A P100 elastomeric respirator filters out at least 99.97% of airborne particles and can block particles 0.3 μm or larger. Elastomeric respirator requires a surgical mask over the exhalation valve if used over a sterile surgical field.

##### Powered air purifying respirators (PAPRs)

are composed of a face mask/hood and a separate fan/motor/filter unit which is typically located on a belt or pack. Similarly, Controlled Air-Purifying Respirators **(**CAPRs), a type of PAPR also deliver fresh, filtered air via a helmet based unit, thus eliminating the hose and replacing it with an electrical wire to a battery pack. These units actively circulate and filter air around an individual’s face. A powered positive outflow of air from within hood/mask is created and can block 99.97% of airborne particles 0.3 μm or larger. These are often unavailable in Canada and require training for safe donning and doffing.

Several publications recommend PAPRs for use in extremely aerosolizing procedures of the airway, lung, sinus oropharynx and skull base surgery [32, 60, 62, 63]. The potential advantages of PAPRs include the conservation of PPE including N95 respirators [64]. Furthermore, while early concerns of potential particulate transfer from HCP using PAPRs to the nearby environment were considered, these have not been born out in experimental and clinical research studies [65, 66]. PAPRs also may have the potential to offer improved comfort for staff operating in high risk areas. It has been demonstrated that PAPRs are valued by end users as being more comfortable than N95 respirators [32, 62, 67]. They allow low breathing resistance and provide a high level of protection [67]. Furthermore, overheating and fogging of eye protection (face shield/visor) as may be seen with other respirators (eg. N95, N99) is generally not an issue with PAPRs due to the constant fresh air flow over these regions.

Additionally, PAPRs are a viable option for staff who are unable to wear an N95. This has been evaluated to be an issue in up to 15–20% of staff members who are unable to wear N95 masks safely [61]. For those who are not N95 fit-tested or those who cannot shave their facial hair due to religious reasons, PAPRs may be used [64].

Although there is some evidence that PAPRs may provide further protection when compared to N95 masks, it is unclear whether this translates into a ‘real life’ benefit in terms of transmission prevention as compared to standard N95 respirators with facial/eye protection. In a recent article that appeared in the Canadian Journal of Anesthesia, Wong, et al. emphasizes the use of PAPR/CAPRs to provide staff with increased protection during AGMP in the OR [67]. A different study evaluating self-contamination during doffing as well as discomfort levels with the use of N95 respirators versus PAPRs showed providers had few problems and that PAPRs were rated with a higher level of protection, than other forms of PPE [68]. The study further highlights potential benefits of PAPRs over N95 with respect to “breathing difficulty, suffocation, heat stress, and fogging-up glasses” with PAPRs and assisted doffing were generally associated with fewer problems and were rated highest. A different study found that experimental exposure of influenza virus to HCPs had breached N95 respirators in 10% of exposures, however those wearing PAPRs did not have any breakthrough exposures [69]. While this evidence demonstrates that PAPR based systems are likely to be better tolerated, especially in certain situations, and that they are safe in the operating room setting, it remains likely that N95 respirators and equivalents provide a comparable level of protection in the majority of situations and individuals.

#### Eye protection

currently there is no explicit standard for eye protection against SARS-CoV-2 and includes face shields, closely fitted wrap-around safety glasses (goggles), and visors. Prescription eyeglasses do not provide adequate protection [70].

#### Face shields

Face shields are an important consideration to protect the N95 respirator or mask from droplet contamination in addition to eye protection and allow for the safe reuse of the mask with multiple patients. At the completion of the case, the face shield can be easily disinfected. On the other hand, there is evidence in the scientific literature that supports the use of goggles over face shields in order to provide greater protection from aerosols [60]. A simulated study demonstrated that face shields were only 23 to 68% effective at blocking aerosols and that their efficacy decreased with exposure time, however, face shields provide adequate protection from droplets which are considered the main mode of transmission of COVID-19 [71].

#### Gloves

surgical single-use gloves should have long cuffs reaching well above the wrist to cover any exposed skin.

#### Gown

Level 1 gown is an isolation gown which has no fluid resistance used in minimal risk situations. Level 2 gown is a disposable or washable gown that has some fluid resistance, long-sleeved, cuffed, and back is covered used in low risk situations. Level 3 gown is used in moderate risk situations and provides a barrier to larger amounts of fluid than a level 2 gown. Level 4 gown is used in high risk situations and prevents fluid penetration for up to 1 h and may prevent virus penetration for up to 1 h.

#### Hair and shoe covers

The World Health Organization (WHO) does not make any recommendations for the use of shoe cover in the care of COVID-19 patients and the CDC states that shoe or boot covers are not recommended at this time for personnel caring for patients with COVID-19. There is no specific recommendation for hair cover for the care of COVID-19 patients, however, given the operating room setting, all personnel will be wearing hair cover during surgery.

### Viral transmission

The primary mode of transmission of SARS-CoV-2 appears to be via respiratory droplets followed by contact with contaminated surfaces. Although a recently published commentary supported by 239 scientists reports studies supporting a possible mode of airborne transmission [72]. This is an area of much controversial at the time of writing. There have also been case reports of transconjunctival and fecal-oral transmission [73]. Furthermore, it is important to recognize that asymptomatic - and in particular pre-symptomatic - COVID-19 patients may be highly contagious as well. Asymptomatic infections have been more commonly identified in the pediatric population [74], however, there have been some adult cases as well [75].

Although the transmission of SARS-CoV-2 is not yet fully understood, for AGMPs such as endotracheal intubation, there is consensus that N95 respirators offer better protection than surgical masks [76]. During the initial phases of the pandemic, given the uncertainty of disease transmission, extra precautions were implemented to protect HCPs. Anecdotal accounts regarding high rates of infection amongst Otolaryngologists have resulted in great concern regarding sinus and skull base surgery specifically [77]. Patel et al. raised concerns that N95 respirators were not sufficient to mitigate the transmission of the SARS-CoV-2 virus and rather recommended the use of PAPRs for all operating room team members [77, 78]. However, other studies report that some of the early HCP infections in Wuhan may be related to the lack of PPE and education about its correct use [79]. Many experts recommend assuming that every patient is potentially infected with COVID-19 until proven otherwise [77, 80]. However, this assumption should be guided by the rate of community transmission. Over the intervening months since the onset of the pandemic and as more is known about SARS-CoV-2, it is apparent that some of the initial recommendations can be eased for certain patients as we resume scheduled surgeries [81]. One can argue that in a low to very low prevalence setting, assuming that every patient is COVID-19 negative unless suggested otherwise should be applied.

### Appropriate PPE

The determination of appropriate PPE level is dependent on a patient’s COVID-19 status and the risk of aerosolization caused by the surgical intervention. For patients known to be actively infected with COVID-19 and surgery must be conducted on an emergent or urgent basis, maximal PPE should be used during all interactions [59].

A systematic review investigating the effectiveness of medical masks to N95 respirators in preventing laboratory confirmed viral respiratory illness in HCPs found low certainty evidence that suggests they offer similar protection against these infections including coronavirus in HCPs during non-aerosol-generating care [82].

There is limited information in the literature currently regarding the aerosolization risks during endoscopic sinonasal procedures such as septoplasty, endoscopic sinus surgery, or skull base surgery. In a simulated study, Workman et al. demonstrated that no droplets were observed using cold non-powered instruments or with the use of microdebrider [83]. The use of high-speed drilling may result in higher risk of droplet contamination due to the increased airflow velocities. In another cadaveric study, the authors performed simulated sinus surgery in fluorescein coated nasal/sinus mucosa and found limited droplet spread using powered instrumentation including a drill if a concurrent suction was used [84]. A prospective cohort study by Taha et al. assessed the efficacy of a specific institution protocol in preventing transmission of COVID-19 to HCPs during otolaryngology care in a high prevalence setting [85]. All health care providers were required to wear p100 filter respirators, as well as eye protection, surgical gowns and gloves. In a five-week period, 152 endoscopic sinonasal, skull base, and open airway procedures were performed. Despite 17.1% of these patients tested positive for SARS-CoV-2, no HCP tested positive with this protocol. This demonstrates the effectiveness of proper PPE use in the protection of HCPs.

While the appropriate PPE for surgery in the era of COVID-19 has been debated, many sources have advocated for a PAPR when surgery is necessary in AGMPs. However, this level of PPE is often unavailable within Canada, and comes with significant challenges for safe donning and doffing. For longer surgeries, PAPRs may offer benefits such as increased user comfort and reduced fogging. Taken together, current evidence suggests that endoscopic sinus surgery with cold unpowered instruments and microdebrider has a low risk of generating aerosol compared to a higher risk when using a drill. Please see recommendations in Table 2 for guidance on PPE during rhinologic surgery.

**Table 2:** Recommended PPE for Rhinologic Cases

Case Type	Respiratory PPE	PPE
Endonasal septoplasty (with no drilling)	Surgical mask	Eye protection or face shield, surgical gown, gloves
Endoscopic sinus surgery (with no drilling)	N95 or surgical mask	Eye protection and/or face shield, surgical gown, gloves
Endoscopic sinus surgery (with drilling)	N95, P100 respirator, PAPR (optional)	Eye protection and/or face shield, surgical gown, gloves
Endoscopic skull base surgery (with drilling)	N95, P100 respirator, PAPR (optional)	Eye protection and/or face shield, surgical gown, gloves
Intranasal thermal use	N95	Eye protection and/or face shield, surgical gown, gloves

### Education and training

Training on proper donning and doffing of PPE for all HCP is highly recommended to ensure safety of all members of the health care team. Improper doffing can be a high-risk procedure for healthcare workers [80]. A donning and doffing buddy system can also be considered for healthcare providers when managing COVID-19 positive or for those patients under investigation. Furthermore, proper hand hygiene before putting on and after removing PPE, including gloves is an essential component of preventing transmission of any pathogens to bare hands during the removal process [86].

The AAO-HNS also recommends that surgeons consider performing a “PPE timeout” in addition to the traditional surgical timeout prior to the initiation of any case in the operating room [59]. The incorporation of a “PPE timeout” will help to ensure that all team members have a unified understanding of the risks of possible transmission and the importance of the precautions.

## Specific rhinology and Skull Base procedure considerations

### Transnasal Septoplasty

#### Pre-operative considerations

Pre-operative planning is important for exposure reduction to operating room staff during septoplasty. General anesthesia with endotracheal tube intubation is recommended over neurolept anesthesia or the use of laryngopharyngeal mask to decrease the potential risk of aerosolization by the Canadian Anesthesia Society [87].

Preparation of the nasal cavity for septoplasty surgery can vary greatly among surgeons. It is recommended to apply pledgets to the nasal cavity after intubation to reduce the potential risk of aerosolization. As an alternative, one may consider the use of hand-held sprays [88]. The application of a nebulizer may increase the production of droplets or aerosols [89, 90], however, it is uncertain if these droplets can contain viral particles [91, 92]. To prevent potential risk, the use of powered aerosol anesthesia should be avoided.

#### Intra-operative considerations

In a cadaveric simulation of endonasal procedures, Sharma et al. found no evidence of fluoroscein droplet generation during septoplasty [84]. When injecting the septum for hemostasis and hydrodissection, care should be taken to ensure the injection remains submucosal to avoid the theoretical potential of aerosolization. Some consideration for intranasal irrigation with iodine can be considered (See Section 4.2.2).

Nasal suctioning with an 8-French Frazier suction was evaluated by Workman et al. in a cadaveric model evaluating for aerosolization, they found no fluorescein-stained droplets observed [83].

In taking down a septal spur, care should be taken given the anterior location of this work. Some surgeons employ powered methods including the high-speed septoplasty burr. Workman et al. identified endonasal high speed drilling to be highly aerosol generating [83]. The use of an osteotome with mallet may be a preferred technique considering it has a lower risk for droplets to be aerosolized, keeping in mind droplet potential with hammering [84].

For intraoperative hemostasis, epinephrine soaked pledgets are preferred. Thamboo et al. found electrocautery to be aerosol generating with higher current levels associated with more significant particle aerosolization [93]. Moreover, Ishihama also found aerosolized blood in operating room air vent filters after head and neck surgeries utilizing electrocautery [94]. For intractable arterial bleeding off the maxillary crest, bipolar cautery use with accompanied suction is recommended.

Closure should not impart any increased risk for aerosol generation. Packing that the patients can remove themselves at home or no nasal packing may be ways to mitigate aerosol generating potential risk of packing removal by HCPs.

#### Post-operative considerations

Care must be taken in the post-operative care of patients having undergone septoplasty in order to prevent exposure to COVID-19. The main concern is removal of nasal packing/stent regardless of the method used by individual surgeons. The physical act of packing removal may result in droplets from the nose being expelled into the proximity of the person removing the material from the nose. A cough or a sneeze may also be induced, resulting in droplet formation and low-level aerosolization of potential viruses.

One option is for patients to remove their own packing at home. This provides the safest environment for packing to be removed as it does not expose the physician, nurses or staff to potential pathogens. It is recommended that patients removing their own packing to do so in the presence of at least one other person to ensure adequate safety.

If packing is to be removed in the office setting, we recommend a mask be worn by the patient over the mouth to reduce droplet and aerosol spread [83, 95]. We would also suggest that post-operative suctioning or debridement of the nasal cavity not be performed for the same reasons. We recommend appropriate PPE to be worn by HCPs as per local health authority guidelines (see Section 5.0).

Patients may proceed with high volume irrigation post-operatively to flush any clots and mucous from the nasal cavity.

### Thermal procedures in the nose

The heat generated at the tip of an electrocautery instrument ranges between 400 and 600 °C and vaporizes tissue. In a 2020 cross-sectional study, Carr et al. demonstrated the effect of electrocautery settings on aerosolization during tonsillectomy [96]. The group compared monopolar cautery at 12 watts versus 20 watts used in a pediatric tonsillectomy and reported a statistically significant difference in particle number concentration. Airborne particle concentration during tonsillectomy was over 9.5 times higher at the higher setting [96]. Pillinger et al. demonstrated that using a suction clearance device reduces the amount of surgical smoke reaching the surgeon’s mask during thyroid or parathyroid surgery, as measured with an aerosol monitor [97]. Gao et al. investigated the performance of different respiratory protective devices (RPDs) in protecting operating room (OR) personnel from surgical smoke exposure [98]. The group evaluated the efficacy of surgical masks (SM), N95 surgical mask respirators (SMR), and N100 filtering facepiece respirators during surgical cautery dissection of animal tissue in a simulated OR chamber. Particle concentrations during the dissection were measured both inside and outside of the OR personnel’s RPD with a particle size spectrometer. The study concluded that SMs do not provide measurable protection against surgical smoke. SMRs provide improved protection over SMs, but that N100 FFRs offered a significant improvement in protection against surgical smoke compared to SMRs. Workman et al. have recently confirmed this in a cadaveric model where significant aerosol was generated that was a finer particulate than that seen with high-speed instrumentation [99].

Although the theoretical possibility of COVID-19 transmission through electrosurgical smoke exists, actual documented cases of pathogen transmission are rare and limited to non-respiratory viruses. In regards to the potential for transmission of viral particles in this generated aerosol, one study from 1991 has demonstrated viable human papillomavirus in this plume, but note should be made of the extremely high concentration of virus in the ablated papilloma tissue [100]. Hepatitis B DNA has been identified in laparoscopic surgery cautery plume, but the viral cells were not viable [101, 102]. Similarly, an animal study showed HIV cells were identified in aerosols from high drilling instrumentation, but no viable cells were identified in high temperature cautery plume [101].

A review of thermal and laser plumes in the operating room has shown that a proper local exhaust ventilation system in the OR along with use of N95/100 respirator were the most effective in preventing exposure to OR personnel to toxic and biological particles in the plume [103]. They also found PAPR shows no added protection from smoke exposure compared to surgical mask or N95 respirator. The most effective tool shown to decrease the level of electrocautery flumes reaching the operator’s mask was the use of a suctioning device within 2 in. from the smoke source.

Furthermore, the type of cautery device used may also affect the quantity of aerosolization generated. A recent study performed by Smith & Nephew found the Coblation wand radiofrequency device generated 250-fold less aerosol particles than a standard monopolar cautery wand in animal models [104]. Bipolar cautery can also potentially decrease risk of aerosolization compared to monopolar cautery due to lower voltage setting and targeted tissue thermal lesions. In regard to nasal surgery, there is no general consensus or high level of evidence supporting one particular device being superior in safety. The surgeon and their institution may want to consider factors such as the safety, cost, ease of use and availability when selecting the appropriate cautery device to use in nasal procedures.

#### Anterior epistaxis

Patients presenting with epistaxis can be challenging to manage and safety is of highest importance to decrease risk of respiratory and blood-borne pathogen transmissions. Multiple studies have shown that physicians treating patients with epistaxis were visually identified to have blood splatters on their protective equipment [105–107]. The risk of blood and droplets are further heightened if coughing or sneezing is initiated. Simple use of a surgical mask covering a patient’s mouth or use of a modified mask covering the nose but creating a small port for nasal access has shown to significantly decrease risk of droplets and aerosols generated [83].

In the clinical setting, we recommend utilizing the following strategies that decrease risk of droplet or blood aerosolization. The physician should wear full protective equipment during the procedure including surgical mask, eye protection, and gown. The patient should be instructed to cover their mouth with a mask throughout the procedure and instructed to warn the physician if they feel a sneeze coming. Minimizing proximity to the patient’s face during the procedure is recommended and may be improved with use of video endoscopy tower systems if available. In cases of localized anterior epistaxis, particularly in Little’s area, a decongestant or a use of vasoconstrictor topical medication on a pledget or sponge can be used to temporize the bleeding. Avoiding (especially powered) nebulized medications to decrease theoretical risk of aerosolization of nasal secretions may be beneficial. Silver nitrate, as opposed to electrocautery is ideal to minimize cautery plume. If possible, keep a suction close to patient’s nare to minimize aerosol spread and capture any blood or viral particles. This can be done by the patient holding the suction device such as a Yankauer near their nose or with the help of an assistant. If packing is required, it would be ideal to use dissolvable packing to eliminate the similar risks of packing removal at the next encounter. For non-dissolvable packing options, avoid ribbon gauge packs as they are more tedious and time consuming to complete, instead, if appropriate, consider a simple lubricated Merocel(™) or Rapid Rhino(™) nasal pack which requires a simple one push technique and easier to remove in the subsequent visit.

#### Posterior epistaxis

Similar strategies to anterior epistaxis should be considered to minimize risk of aerosolization and transmission of blood-born and respiratory pathogens. For posterior packing, avoid traditional foley technique as it requires examining the oropharynx for proper placement which can increase risk of aerosol production. A quicker option with an Epistat(™) balloon, long Merocels(™) or Rapid Rhino (™) bilaterally may be optimal if appropriate. If a patient requires surgical exploration, consider surgical clips for sphenopalatine ligation over cauterization if the patient is COVID-19 positive. Otherwise, a point of care risk assessment can be performed to determine which approach is best in the surgeon’s experience. If cautery is utilized, use suctioning in the contralateral nostril when cautery is used. Furthermore, it would be prudent to consider bipolar cautery over monopolar as it limits the area of thermal injury and decreases risk of unnecessary aerosolization from normal nasal tissue. In cases where the patient is COVID-19 positive, personal protective equipment is limited or surgical expertise is not readily available, endovascular embolization techniques could be considered as an alternative to control posterior bleeds.

#### Turbinate reduction

Various methods of turbinate reduction have been described including outfracturing, cauterization, submucosal resection, radiofrequency ablation, cryotherapy and microdebrider technique. The indication and the risk/benefit ratio of the technique for turbinate reduction should be weighed against the potential for aerosol generation in each individual patient. During any potential aerosol-generating procedure, a suction should be used in the contralateral nare placed in the nasopharynx. Prior to any surgical procedures of turbinates, a vasoconstrictor applied topically such as epinephrine or oxymetazoline on pledgets and injecting the turbinate with local anesthetic should be considered to decrease risk of bleeding during the procedure. Radiofrequency technique may provide the least risk of aerosolization and thermal injury to surrounding tissue. Performing the reduction in the OR under general anesthetic allows for control of the patient’s airway and will limit the potential for patient sneezing and bleeding. We suggest not using any post procedure stents or packing which may be needed to be removed in the post-operative visit (see Section 6.1). Consider using dissolving packing material such as surgicel, gelfoam or topical thrombin (Floseal(™)) to act as a hemostatic agent in the post-operative setting.

### Endoscopic sinus surgery (ESS)

There is a paucity of evidence on aerosolization and droplet spread during endoscopic sinus surgery. Recently, Workman et al. simulated aerosolization events performing a variety of endoscopic endonasal procedures and found that non-powered cold instrumentation and microdebrider use did not generate detectable aerosols [83]. It is not known if the microdebrider may generate droplets/aerosol if the suction port becomes obstructed/plugged as is occasionally the case during ESS. In another cadaveric study, the authors investigated fluorescein droplet and splatter patterns resulting from commonly performed endoscopic endonasal procedures as well [84]. No fluorescein droplet spread was observed in the measured surgical field in any direction after nasal endoscopy, microdebrider-assisted turbinoplasty, and ESS with non-powered instrumentation. In addition, drilling of sphenoid rostrum with a cutting burr (4 mm), drilling of frontal beak with a diamond burr (4 mm), drilling of sphenoid rostrum with a diamond burr with concurrent suction (4 mm), drilling of frontal beak with concurrent suction, ultrasonic aspirator on left sphenoid sinus, ultrasonic aspirator on right frontal sinus, and external activation of the ultrasonic aspirator also did not result in droplet spread outside of the nasal cavities [84]. Conditions that resulted in limited contamination of droplet sizes less than 1 mm include ESS with microdebrider (2 droplets within 10 cm of cadaver head) and powered drilling of sphenoid rostrum (8 droplets within 12 cm of cadaver head) and frontal beak drilling (5 droplets within 9 cm of cadaver head). The addition of concurrent suction use while drilling eliminated the contamination. Running a contaminated drill outside the nasal cavity resulted in gross droplet spread, as expected, indicating that the soft tissue confines of the nasal cavities function as a barrier in preventing droplet splatter. Taken together, these studies demonstrated only limited droplet spread using powered instrumentation endoscopically which can be mitigated by the concurrent use of suction.

Lastly, with respect to rhinology and endoscopic skull base cases in particular, there is the opportunity to have suctions intermittently and/or continuously within the surgical field, in an attempt to limit escape of potential aerosols from the sino-nasal cavity (see Section 6.4.6.1). Workman et al. found in their experimental study that having a suction in the nose seemed to reduce the detectable aerosols that escaped, suggesting there is a benefit to this method of creating a local negative pressure environment *within* the surgical field [99]. There are anecdotal descriptions of suction catheters placed in the nasopharynx throughout the case, continuously suctioning aerosols posteriorly.

#### Pre-operative preparation

Local anesthesia injection, throat pack, and Kennedy nasopharyngeal packing to be used as per surgeon routine. The placement of a 12 French catheter suction along the inferior turbinate on one side placed in nasopharynx and stapled to the drape may help mitigate droplet spread (see Section 6.4.6.1). The same can be introduced into nasopharynx via the oral cavity and secured at the oral cavity with an Op-site. If only operating on a single side, can consider using an adhesive dressing to occlude the opposite nostril around the 12 French catheter suction. Draping to expose only the nostrils and cover the oral cavity will help decrease droplet or aerosol spread during surgery. Pledget use for decongestant application or drip decongestant into the nose during induction may also help in decreasing aerosol generation.

#### Intraoperative practical tips

The use of cold steel instrumentation as much as possible is recommended to decrease chances of aerosolization. Microdebrider use only intranasally, try not to activate contaminated microdebrider outside the nasal cavity. It is important to keep the microdebrider blade in the closed position when it is not in use to help minimize contamination outside the surgical field. If possible, the microdebrider insertion and removal from nasal cavities should be minimized. The use of an irrigation sleeve to help with visualization will help to minimize moving scope in and out of the nasal cavity as well. Surgeons should consider using punches rather than a drill for sphenoidotomy or frontal sinusotomy as much as possible (drilling will be addressed in Section 6.4).

#### End of surgery and post-operative care

Absorbable packing for hemostasis should be used as much as possible to minimize manipulation, removal, and debridement post-operatively in the office or clinic. It is important to remove excess blood and fluid from nasopharynx prior to extubation to reduce aerosolization risks. The patient should start large volume saline irrigation post-operatively to help remove any absorbable packing, clots, old blood, mucous rather than debridement in the office or clinic setting. The area at home where patients perform their nasal irrigation will need to be disinfected after use if being used by other household members. Similarly, other household members should utilize contact/droplet precautions or avoid the area while irrigation is underway.

### Skull Base surgery

#### Background

CoVID-19 emerged in early 2020 as a global pandemic and with it came significant concern and recommendations to help guide surgical practice in the field of endoscopic skull base surgery. Early opinions were based on limited evidence and anecdotal reporting of transmission of disease amongst HCPs. A clear understanding of the facts surrounding the early cases is important as we try to emerge from a near complete global shut down of endoscopic skull base surgery.

#### How did we get Here?

In their original letter to the Editor in Neurosurgery, Patel et al. summarized the evidence they had gathered with respect to COVID-19 and its effects on OR personnel in Wuhan, China [108]. They stated that more than 14 members of the patient care team both within and outside the operating room became infected with COVID-19 after a trans-sphenoidal surgery (TSS) in early January in Wuhan on a patient with mild flu-like symptoms. In addition, a second TSS later in January on a patient with pituitary apoplexy and fever with imaging changes to suggest viral pneumonia was operated on with “appropriate” PPE. During this second case the neurosurgeon and 2 OR nurses wore N95 respirators and the anesthesiologist wore a homemade positive pressure helmet. Within 3–4 days, the surgeon and the 2 OR nurses developed fever and respiratory symptoms compatible with pneumonia. However, no confirmed COVID-19 positive testing was reported. The anesthesiologist did not develop any symptoms and the others all fully recovered.

The group from Stanford also commented on personal communications with Otolaryngologists in Iran and stated that at least 20 Otolaryngologists were hospitalized (COVID-19 swab positive) and 20 more were at home in isolation with no swab results [108]. Media reports from the United Kingdom stated that the British Association of Otorhinolaryngology confirmed 2 consultants were COVID-19 positive and on ventilatory support [109]. Understandably, these very concerning reports led to significant anxiety in the skull base community and quickly, most non-life-threatening endoscopic skull base surgery was halted globally.

The neurosurgical group from Wuhan formulated a rebuttal to clarify the facts surrounding the sentinel case that was being discussed widely in several surgical societies and forums [110]. They stated that one patient underwent TSS on Jan 6th and was diagnosed with COVID-19 13 days later. Notably, none of the medical staff who participated in the surgery were infected with COVID-19. There were 10 nurses and 4 surgeons who were infected and only 4 nurses had direct patient contact with this individual. It was presumed they all became infected during post-operative care as the patient was transferred between 3 different hospital wards after surgery. In addition, they have no data to support the story on the second case of fever and respiratory symptoms in OR personnel wearing appropriate PPE within the neurosurgery group in Wuhan.

To the best of our knowledge after a review of the literature there are no other reports of intraoperative spread of COVID-19 in personnel wearing appropriate PPE following endoscopic skull base surgery.

#### Experimental evidence of high-speed instruments

The literature is rapidly evolving and currently contains 3 objective cadaveric studies exploring droplet and aerosol generation in transnasal surgery [83, 84, 99]. Their collective findings suggest that limited droplet spread could occur during microdebrider use [84], and aerosolization occurs with use of electrocautery and a high-speed drill. However, they also describe a lack of aerosol formation with nasal suctioning, cold surgical instrumentation such as cutting forceps, as well as with the microdebrider, due to a relatively low speed of rotation, and aspiration through its large-bore suction (assuming the suction remain unplugged) [83, 99]. The obvious weakness of these cadaver studies is that they do not assess viral load/content and infectious transmissibility (Also see Section 6.3).

#### Similarities to other aerosol generating procedures in the head and neck region

The early response to concerns about aerosol generation during skull base surgery and possible increased risk of infectivity, in addition to systematic down-scaling of OR activity, has led to a large reduction of performance of skull base surgery. Knowledge about infection prevention and risk mitigation can be gained from within the Otolaryngology-Head and Neck Surgery specialty community and from other specialties who perform procedures in the upper aerodigestive tract. Many regional authorities, including Public Health Ontario, have defined aerosol generating procedures to include tracheostomy, surgical procedures with high-speed instrumentation in the aerodigestive tract, and dental procedures requiring high speed drilling [111]. Parallels can be drawn from these areas.

Early studies have suggested that the lower respiratory tract and not the upper respiratory tract may have the highest viral loads of SARS-CoV-2, suggesting that tracheal or bronchial surgery may be higher risk than procedures of the upper respiratory tract and skull base [112]. Guidance issued for performance of tracheostomy procedures in COVID-19 positive patients suggests the need for negative pressure rooms or in the absence of negative pressure rooms, consideration for high efficiency particulate air filtration (HEPA) air filtration systems as an alternative. Recommendations for protective equipment include the use of PAPRs in addition to fluid repellent disposable gowns, eye protection, and gloves in presumed or known COVID-19 positive patients. As an alternative in the absence of PAPR’s, at a minimum the use of fit-tested N95 or FFP3 masks in addition to the aforementioned measures. In the COVID negative patient, the Canadian Society of Otolaryngology task force on performance of tracheostomy has suggested performance by the most experienced person, the use of N95, and full facial and neck protection, and either 1 or 2 pre-operative tests given the uncertain false negative rate of the test at present [113]. The same group recommended use of PAPR in patients who have unknown or presumed COVID-19 positive status to mitigate risk understanding that not all centres have access to this level of protective equipment.

Although it is not entirely clear which procedures are the highest risk for aerosolization of viral particles, previous studies have shown that electrocautery, drilling, ultrasonic devices such as the harmonic scalpel, and suction irrigation may cause aerosolization of viral particles [114–117]. These types of surgical instrumentation are not unique to skull base surgery and are used in head and neck cancer procedures, otologic procedures, as well as dental procedures.

For management of head and neck cancers, because of the importance of time-sensitive surgery to avoid adverse outcomes such as stage migration and inoperability, there have been relatively fewer reductions in surgical volumes compared to other surgical subspecialties. Recommendations have been variable but include pre-operative testing (in some cases two pre-operative tests or the addition of chest imaging to minimize false negative tests), use of full protective equipment including disposable gowns and caps, N95 respirators and face shields, and preference for the use of negative pressure ORs where possible [118]. Some groups have recommended use of PAPRs as a preference [118]. Creation of barriers to prevent aerosolization of mucosal surfaces has similarly been recommended where possible [118]. Early recommendations from an expert panel suggested that in COVID-19 positive patients requiring urgent surgery, consideration should be given to use of PAPR or at least a single-use N95 respirator with face-shield and/or goggles, gown, and double gloves, while in patients who are COVID-19 negative or low risk, practitioners should consider N95 respirator and eye protection, or if unavailable surgical mask with goggles or face shield, gown, and double gloves [118].

The Royal College of Dental Surgeons of Canada have recently issued guidance for our dental colleagues, who perform similar procedures with powered instrumentation [119]. This advisory body has suggested screening of all patients with routine questions, but does not specifically require COVID-19 testing prior to performance of AGMPs [120]. Based on this screening process, patients are classified as screen positive or negative. For patients screening positive and who are undergoing AGMPs, recommendations for use of PPE include use of fit-tested N95 respirator, gloves, eye protection and face shield and protective gowns. For patients who screen negative and are undergoing AGMPs, use of N95 or surgical mask in addition to gloves, eye protection and/or face shield, and a protective gown has been recommended.

#### Neurotropism of COVID-19

As mentioned above, SARS-Cov-2 is found in high density in nasal and nasopharyngeal tissue. Furthermore, this virus displays neurotropism suspected to occur via a trans-cribriform route which may impart additional risk for surgery in this region [121].

#### Intra-op considerations - maneuvers to reduce likelihood for viral Aerosolization

Anesthesia and other institutional risk mitigation strategies during the COVID-19 pandemic including The Hierarchy of Controls are well covered elsewhere in the literature and should be adhered to while taking into consideration local conditions and guidelines.

Use of negative pressure rooms or HEPA filtration should be considered for COVID-19 positive patients on the basis of availability of resources and discussion with local institutional IPAC, HVAC, and engineering staff (see Section 4.1). Other measures mentioned above such as prepping the operative field including the nasal cavity with (0.5%) Povidone-Iodine are generalizable to expanded endonasal skull base approaches (see Section 4.2.2).

##### Creating a negative pressure zone within the Nasopharynx

**Fig. 1:**
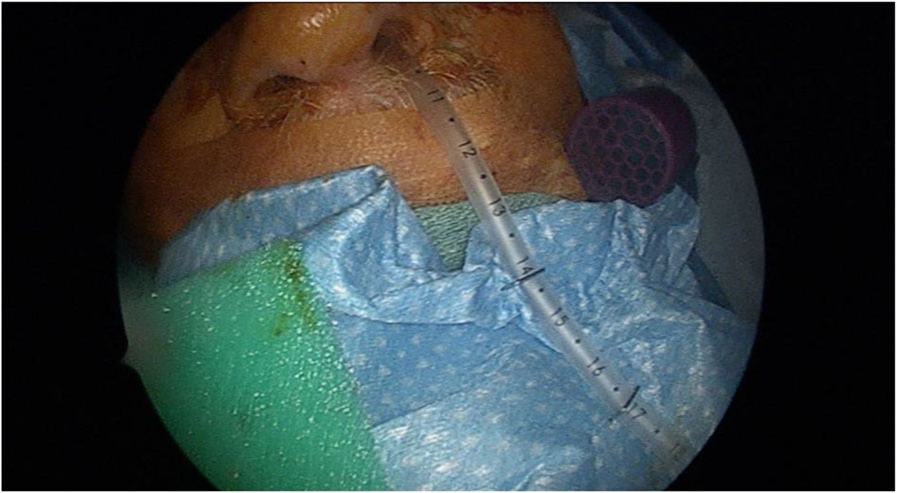
Intra-operative photo demonstrating nasopharyngeal suction catheter stapled to drape and Bufallo filter suction tubing secured to left cheek

**Fig. 2:**
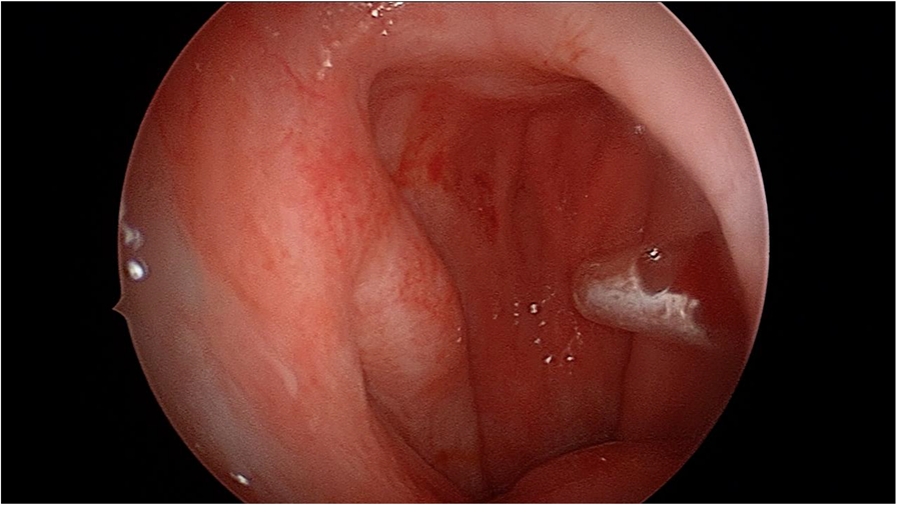
Intraoperative photo demonstrating ideal positioning of nasopharyngeal suction catheter 3–5 mm from posterior pharyngeal wall

As mentioned previously, due to the frequent use of powered instrumentation and electrocautery which may be difficult to avoid, the creation of an aerosol/droplet plume during more advanced endoscopic skull base procedures is a potential significant risk to the surgical team members. One option used by some of the members of the task force has been to direct the airflow away from the nostrils. This may be accomplished through the use of dynamic suctioning while drilling or cauterizing. As well, the use of a flexible endotracheal suction catheter inserted either via the non-dominant nostril, under the inferior turbinate or trans-orally to constantly suction air and fluid throughout the entire surgery is a recommended option. This serves to orient the airflow posteriorly to the nasopharynx and serves to minimize the risk of bioaerosol exiting through the nostrils (see Figs. 1 and 2). Technical nuances include having the suction secured with a stapler to the drapes so that it rests approximately 5–10 mm from the back of the nasopharynx. The suction should be checked periodically to ensure it is not obstructed with clot or debris. If a naso-septal flap is required, it may be best to tuck it into a maxillary sinus so it does not obstruct the suction catheter in the nasopharynx.

##### Other strategies

Additional use of airflow dynamics to minimize aerosol spread include the use of a smoke evacuator (eg. PlumeSafe® Turbo, Buffalo Filter, NY, NY). This may be secured to the cheek or just outside the nostril to evacuate any aerosol/droplet which may have exited the nostrils (see Fig. 1).

Other risk mitigating strategies may include minimizing electrocautery, or reducing the amplitude/current settings (see Section 6.2). Similarly, reducing speed settings of powered rotating instruments such as drills may also be utilized to potentially decrease aerosol generation during endoscopic skull base procedures as appropriate.

##### Barrier options

A recent publication describes the use of a ventilated type mask and laparoscopy trocar as a method to reduce aerosol spread during skull base procedures [122]. Another study proposed the use of a plastic drape suspended over the patient’s head with a smoke evacuator suction placed inside the chamber to create a negative pressure environment in endonasal surgery [123]. Droplets were identified at the tip of the smoke evacuator in cases with prolonged high-speed drilling, leading the authors to hypothesize that this apparatus may effectively capture fluid in the form of droplets or aerosols. Utilization of these types of methods may complicate the facility of introduction and freedom of movement of surgical instruments into the operative field.

Common sense interventions such as minimizing surgical time and bleeding are also recommended especially in high risk patients and communities. Furthermore, surgical education and exposure of trainees to these types of cases should be considered with respect to factors such as available resources and risks to health care worker exposure.

#### Post-operative considerations

To the extent possible, post-operative debridements in the office or clinic should be minimized or avoided altogether. Light packing of the sphenoid sinus or nasal cavity using fully dissolvable materials such as gelfoam, oxycellulose, or starch-based packings should be considered at the conclusion of surgery. Patients should irrigate their nasal cavities with a sterile pressurized saline spray 3–6 times a day for the initial post-operative period; this may be required beyond a month if no debridement is performed in the office or clinic.

Although some sources have advocated for the shift of extended endonasal approaches to intracranial corridors (e.g. pituitary pathology, CSF leak repair), we believe that in the considerable majority of cases, this may not be prudent [28, 124]. When considering patient outcomes as well as risks to the healthcare teams, our expert panel consensus recommends that the trans-nasal route remain as the preferred surgical pathway for the majority of pathologies that have migrated to this route during the pre-COVID era.

## In-office Rhinologic procedures

In recent years, there is a move from OR-based surgeries to in-office procedures in the field of rhinology [125]. These cases include limited endoscopic sinus surgery, limited septoplasty, nasal polypectomy, turbinate reduction, balloon sinus dilation, and eustachian tube dilation procedures. Most of these procedures are performed under direct visualization using a rigid endoscope.

It is reasonable to perform nasal procedures that are non-AGMP in the clinical setting during the COVID-19 pandemic. The following recommendations are based on existing guidelines [126–128] and are designed to help surgeons and staff provide safe but timely care for patients needing scheduled nasal surgery.

### Pre-office visit precautions

A number of pre-visit precautions are necessary to prepare the patient and minimize time the patient spends in the office or clinic. Virtual consult for history and screening should be offered initially with COVID-19 screening questions. Patient information via intake form can be taken over the phone and inputted into electronic medical records. Patients should be provided a phone number to the office or clinic to call upon arrival to control overcrowding of the waiting area. Patients are instructed to come alone unless minor (one parent only) or if a translator or caregiver is required. If sedation is planned then a driver who can pick them up after procedure will be necessary. Office or clinic staff to advise patients to wear mask or face covering when they come to their appointment.

### Patient selection

Careful patient selection is crucial in determining the success of any in-office procedures. This includes anatomy and disease appropriate for in-office surgery, non-claustrophobic or histrionic phenotype, and avoidance of patients at risk for post-operative bleeding (eg unable to come off anticoagulation), and ASA score (American Society of Anesthesiologists) 1–3 level (ASA 3 only if procedure is low risk) patients.

### Pre-operative precautions

Pre-operative COVID-19 RT-PCR nasopharyngeal test should be obtained if appropriate (see Section 3.0). COVID-19 positive patients should not come in for scheduled surgery. COVID-19 screening questions should be repeated at time of arrival. Temperature check and vital signs are taken upon arrival.

### Intra-operative and post-operative considerations

Given the risk of possible aerosol generation in endonasal procedures, the number of personnel in the room should be minimized. All staff present at the procedure are to wear appropriate PPE for the procedure (see Section 5.0). The use of pledgets for topical anesthesia and decongestion locally is preferred over powered atomizers, however manual spray is considered safe. Local anesthesia should be injected as per usual procedure. There is some evidence for the use of Povidone prep pre-operatively (see Section 4.0). The use of Ativan 1 mg sedation can be considered to help keep the patient calm. Tranexamic acid (IV, oral, or topical) can be used to decrease risk of bleeding post-operatively. A closed suction system (wall suction, or filter on suction) should be used. Powered drills that can cause aerosols should be avoided and other instruments can be used as usual (microdebrider does not cause significant aerosol) (see Section 6.3).

Physical distancing in the recovery area should be maintained. There is no current requirement for waiting after the procedure is done to open the door or exit room. After each procedure, however, a full room clean should be performed.

## Conclusions

It is hoped that these recommendations will be of use to otolaryngologists starting to perform rhinology procedures in the operating room and in outpatient settings to maintain safety for both the patient and healthcare providers. They are based on best available evidence and expert opinion at the time of writing. Where good evidence is not available, practical advice has been given. Nevertheless, we urge readers to check with their local health authorities and in particular, infection prevention and control practices, in their local hospitals and communities.

## Data Availability

Not applicable.

## Abbreviations

CDC: Centre for Disease Control
COVID-19: Disease caused by the 2019 novel coronavirus
CSF: Cerebrospinal fluid
Aerosol: Abbreviation for “aero-solution”
AGMP: Aerosol generating medical procedure
Ig: Immunoglobulin
HCP: Health care provider
PAPR: Powered air purifying respirator
PCRA: Point of care risk assessment
PPE: Personal protective equipment
RT-PCR: Reverse transcriptase – polymerase chain reaction
SARS-CoV-2: Severe acute respiratory syndrome coronavirus 2 virus
WHO: World Health Organization
